# Does 3D Phenotyping Yield Substantial Insights in the Genetics of the Mouse Mandible Shape?

**DOI:** 10.1534/g3.115.024372

**Published:** 2016-02-23

**Authors:** Nicolas Navarro, A. Murat Maga

**Affiliations:** * Biogéosciences, UMR CNRS 6282, Univ Bourgogne Franche-Comté, EPHE, PSL Research University, F-21000 Dijon, France; † Division of Craniofacial Medicine, Department of Pediatrics, University of Washington, Seattle, Washington 98105; ‡ Center for Developmental Biology and Regenerative Medicine, Seattle Children’s Research Institute, Seattle, Washington 98101

**Keywords:** 3D geometric morphometrics, multivariate QTL mapping, *Mus musculus*, mandible shape

## Abstract

We describe the application of high-resolution 3D microcomputed tomography, together with 3D landmarks and geometric morphometrics, to validate and further improve previous quantitative genetic studies that reported QTL responsible for variation in the mandible shape of laboratory mice using a new backcross between C57BL/6J and A/J inbred strains. Despite the increasing availability of 3D imaging techniques, artificial flattening of the mandible by 2D imaging techniques seems at first an acceptable compromise for large-scale phenotyping protocols, thanks to an abundance of low-cost digital imaging systems such as microscopes or digital cameras. We evaluated the gain of information from considering explicitly this additional third dimension, and also from capturing variation on the bone surface where no precise anatomical landmark can be marked. Multivariate QTL mapping conducted with different landmark configurations (2D *vs.* 3D; manual *vs.* semilandmarks) broadly agreed with the findings of previous studies. Significantly more QTL (23) were identified and more precisely mapped when the mandible shape was captured with a large set of semilandmarks coupled with manual landmarks. It appears that finer phenotypic characterization of the mandibular shape with 3D landmarks, along with higher density genotyping, yields better insights into the genetic architecture of mandibular development. Most of the main variation is, nonetheless, preferentially embedded in the natural 2D plane of the hemi-mandible, reinforcing the results of earlier influential investigations.

Geometric morphometric methods based on landmarks offer a convenient statistical framework to conduct quantitative genetic analyses of the shape of complex morphological structures such as the skull and mandible (Klingenberg 2010). Among model systems in the genetics and development of complex traits, the mouse mandible is probably one of the most extensively used (Atchley *et al.* 1985; Atchley and Hall 1991). The primary reason for this success is its intermediate level of complexity (Klingenberg and Navarro 2012) between rather complex models, such as the skull (Hallgrímsson *et al.* 2014), and simpler models, such as the *Drosophila* wing (Dworkin *et al.* 2011; Debat *et al.* 2009) or other insect appendages (*e.g.*, Khila *et al.* 2009; Emília Santos *et al.* 2015; Prud’homme *et al.* 2012). One historical reason, and probably a major constituent of that success, is the relative flatness of this bone, allowing rudimentary 2D imaging techniques to be used effectively.

Genetic architecture, imprinting effects, integration, and modularity of the mouse mandible have been investigated in fairly high detail using geometric morphometrics (Klingenberg *et al.* 2001, 2004; Leamy *et al.* 2008; Suto 2009; Boell *et al.* 2011, 2013; Boell 2013; Boell and Tautz 2011). One common assumption in the majority of these studies is the approximation of the mandible to a 2D shape based on a photograph of either the medial or the buccal sides. The 2D imaging of 3D shapes is well known to incur information loss and errors due to object projection (see Cardini 2014, for a review of this source of error). This flattening may represent a major factor of variation in the sample because of its intricate interaction with the positioning of the object and its shape itself. Nonetheless, for years it has been a common practice in the community to collect data in 2D, mainly because of technical availability and feasibility as well as processing speed compared to 3D data. Despite potential consequences, the 2D projection error inherent to this practice has rarely been explicitly assessed in morphometric protocols compared to other kind of digitization and observer errors that are routinely controlled (*e.g.*, Muñoz-Muñoz and Perpiñan 2010; Yezerinac *et al.* 1992). For the mandible, the sole study that we are aware of reports that 2D approximation is actually accurate for marmot data (Cardini 2014). With the increased availability and reduced cost of both surface and volumetric high-resolution imaging, it is becoming more and more feasible to conduct studies using 3D landmarks, thus reducing the risks of issues related to artificial 2D flattening.

At the same time, in genetics, there is growing interest in deciphering the genetic architecture of within-population variation and local adaptation using genome-wide association studies in either natural populations or outbred stocks (Mott and Flint 2013; Flint and Eskin 2012). In such populations, variants segregate at variable frequencies and linkage disequilibrium is in the order of a few dozen kb (Yalcin *et al.* 2010), and this molecular variation needs to be captured. Nowadays, dense SNP maps obtained from genotyping arrays that are commercially available, or from diverse genotyping-by-sequencing techniques such as whole-genome resequencing (Huang *et al.* 2009), RAD-seq (Baird *et al.* 2008; Peterson *et al.* 2012; Miller *et al.* 2007), multiplexed shotgun sequencing (Andolfatto *et al.* 2011; Cande *et al.* 2012), or targeted capture (Jones and Good 2016; Linnen *et al.* 2013; Olson 2007; Chevalier *et al.* 2014; Hodges *et al.* 2007; Gnirke *et al.* 2009), may yielded thousands to millions of SNPs even in species lacking a reference genome (Elshire *et al.* 2011). This structure of molecular variation requires the use of large sample sizes that are in the order of a few thousands in the best case scenario of average minor allele frequencies (*e.g.*, Valdar *et al.* 2006; Navarro and Klingenberg 2007). Failure to reach these high sample sizes will undoubtedly result in difficulties with reaching significance and being able to make decisions between noise and signal in associations (Ledur *et al.* 2009). These studies may finally come up with mixed results, mapping the main players but accounting only for a low fraction of the total genetic variance, suggesting a large amount of missing heritability (*e.g.*, Pallares *et al.* 2014).

Whether the imaging modality is 2D or 3D, the process of acquiring landmark coordinates has remained predominantly the same over the last 30 years, *i.e.*, tedious, manual expert annotation of the anatomy. Moreover, the third dimension presents unique challenges (*e.g.*, dealing with the accurate projection of 3D structures on a 2D medium like a computer screen and/or artifacts relating to the orthographic or perspective rendering of the specimen), and the amount of time required to access the accuracy of landmarking (*i.e.*, making sure the selected landmark is actually located on the specimen, not an artifact of the 3D rendering angle) clearly counterbalances the gains by a dramatic cost on the actual feasible sample size. Landmarking in 3D requires 10–60 sec/landmark depending on the expertise and software used (Bromiley *et al.* 2014). These numbers are also in the order of time required for the digitization of a complete mandible in 2D. Modern phenomics need large sample sizes to follow high-throughput genomic technologies and questions of modern genetics (Houle *et al.* 2010). A variety of alternatives to manual expert annotation exist or are currently under development depending on the field and the imaging modalities used (Perakis *et al.* 2014, 2010; Liu *et al.* 2008; Aneja *et al.* 2015; Guo *et al.* 2013). For instance, attempts have been made to annotate landmarks on CT scans of new specimens using a machine learning algorithm applied to an initial training set created by experts (Bromiley *et al.* 2014), or using registrations of whole surfaces or volumes (Rolfe *et al.* 2011), sometimes coupled with multiple atlases, allowing precision comparable to manual editing (Young and Maga 2015). Most approaches are actually based on the dense registration of whole surfaces or volumes and therefore encapsulate homologies at the level of the whole structure. In such phenotyping protocols, constraining the registration of the whole structure with some expert annotation improves point correspondence by ensuring homologies of some anatomical features (McCane 2013). The semilandmark approach (Bookstein 1997; Gunz *et al.* 2005; Gunz and Mitteroecker 2013) employs such points on curves and surfaces for which homology conditions are relaxed. These landmarks do not have a one-to-one correspondence but quantify anatomical regions where precise manual annotation is not feasible or possible. The technique uses true landmarks to anchor the homology, and the optimal placement of these points is obtained by sliding them locally on the surface until either the Procrustes distance or the Bending energy is minimized (Gunz and Mitteroecker 2013).

Here, we want to revisit some of the QTL studies of mandibular shape in mice using landmarks acquired in 3D. We chose the mandible as our structure of interest because it is a well-studied model system, on which several QTL mapping studies of geometric shape have already been conducted in the past in inbred intercrosses (Klingenberg *et al.* 2001; Leamy *et al.* 2008). Since none of these studies have associated 3D data for their samples, we turned to a new mouse backcross between A/J and C57BL/6J that we recently reported (Maga *et al.* 2015). We wanted to reassess mandibular shape genetics using this new dataset and 3D phenotyping, and evaluate the gain of information (if any) from the third dimension, given that mandible flattening seems at first an acceptable compromise for large-scale study. As a secondary goal, we wanted to increase the phenotypic coverage of the mandible using a template of semilandmarks that was tied into the already collected expert landmarks and assess whether any further benefit was obtained from dense phenotypic coverage.

## Materials and Methods

### Experimental design and statistical shape analysis

All aspects of the experimental design, genotyping, the rationale for mapping shape loci using multivariate techniques, and a complete development of the multiple QTL mapping approach used are detailed in an open paper (Maga *et al.* 2015). Briefly, skulls of 433 (A/J×C57BL/6J)×A/J 28-day-old individuals were microCT scanned at 18 mm spatial resolution, and genotyped from liver tissue at 882 informative autosomal SNPs using the Illumina medium density linkage panel. After phenotyping and removing six incomplete specimens (see below for specific details relative to the mandible), a full generalized Procrustes analysis (Dryden and Mardia 1998) was performed on these 3D landmarks using the R/Morpho package (Schlager 2015a), and then multivariate shape QTL mapping was done using the R/shapeQTL package (Navarro 2015) of R statistical software (R Core Team 2015). All animal protocols were approved by the University of Washington’s Institutional Animal Care and Use Committee.

### 3D phenotyping: manual landmarks and simulated 2D phenotyping

**Figure 1:**
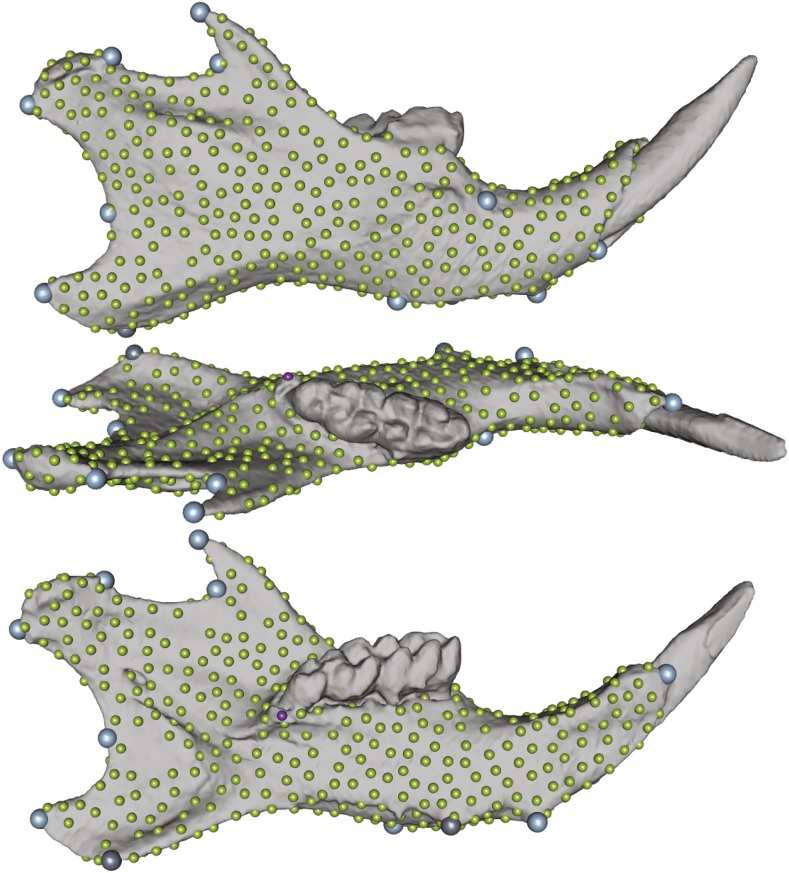
Mandibular 3D surface, landmark, and semilandmark templates. Light blue and gray dots are manual 3D landmarks. Small green dots represent the semilandmark template. The two gray landmarks and the small purple dot were used as the initial reference plane for 2D flattening prior to optimization.

Thirteen 3D landmarks from the right mandible (Figure 1) were acquired twice from 3D renderings of original image stacks of the complete skull using 3D Slicer (Fedorov *et al.* 2012, http://www.slicer.org). The sets were averaged as the best estimate of the landmark location. These landmarks correspond to the classical set of ∼15 landmarks used in previous studies of mouse mandible genetics (Atchley *et al.* 1985; Klingenberg *et al.* 2004; Leamy *et al.* 2008). We initially acquired more landmarks but we found some gross or systematic errors on several of them, which were consequently removed. On the remaining set of 13, no systematic error between landmarking sessions was found ($F_{32,821}=1.28,p=0.14$), and the percentage of measurement error was 4.38% and ranged from 2.6–7.55% per landmark.

For a fair comparison with existing results based on 2D imaging, and to better evaluate the gain from the third dimension independently of the gain of denser genotyping, we artificially flattened the mandibular 3D landmarks. To do that, we first aligned each mandible to its main axes, then chose three landmarks to start (dark gray and purple landmarks in Figure 1): the lower point of the angular process, the lower end of symphysis, and an additional landmark (purple landmark) at the top of the inner ridge meeting the molar alveolus. Positions of the first two were refined iteratively based on the innermost vertex of the mesh along the normal to the plane they defined with the third landmark. These three landmarks defined an approximate natural plane on which the mandible would lay. All 13 landmarks were then orthogonally projected according to the normal to this plane.

### 3D phenotyping: semilandmarks

Right mandibles were segmented from the articulated heads. Image voxel resolution was reduced from 18 mm to 36 mm to make further image processing and computations more feasible. We applied a global threshold to remove nonbone material followed by a watershed algorithm to fill any gaps, since watertight meshes are critical for generating semilandmarks on the bone surface. A 3D Gaussian filter with σ=0.2 was applied to reduce the noise in voxel data. Along this process, six specimens were identified with incomplete mandible scans and removed from the analysis.

Because the manual landmarks were annotated on the original full-resolution, articulated mouse heads, small differences may exist between the 3D surface and the 3D landmarks due to the image processing pipeline employed. Therefore, we back-projected the averaged landmarks onto the mandibular surface based on the shortest Euclidean distance between the landmark and the 3D mesh. This ensured that the manual landmarks also existed on the hemi-mandible meshes generated for this analysis.

A template of uniformly distributed semilandmarks was generated using poisson-disk sampling on the closest individual to the mean shape of 3D landmarks. As an alternative, targeted templates using curves and patches may have been developed to focus on specific anatomical features like ridges on the surface (*e.g.*, masseteric ridge) or borders (Swiderski and Zelditch 2013; Anderson *et al.* 2014), but our aim was to model the bone surface densely. Points lying on the incisors or molars were also manually identified and removed from the template. In the end, we retained 579 semilandmarks (green landmarks in Figure 1) in addition to our 13 expert-annotated landmarks. This template was transferred onto new samples by thin-plate splines based on these 13 landmarks.

Semilandmarks were further slided based on the bending energy and back-projected on the actual mesh surfaces after the sliding relaxation (Gunz *et al.* 2005). Both the expert-annotated landmark and the semilandmark data were subjected to two independent full GPA. The described procedure made use of the vcgSample function from the Rvcg package (Schlager 2015b) and the closemeshKD, placePatch, and 3Dslider functions of the R/Morpho package (Schlager 2015a).

### Shape QTL mapping

The effect at the locus *l* was estimated using the multivariate linear model $y_{i}|M_{i}∼N_{q}\left(\mu +\sum_{c}x_{ic}\beta_{c}+\sum_{j}p_{ij}\beta_{j},S\right)$, where $x_{ic}$ is the value of the covariate *c* and $p_{ij}=Pr\left(g_{i}=j|M_{i}\right)$ is the probability of the QTL genotypes given the flanking markers *M* for individual *i*. These probabilities were computed using R/qtl (Broman *et al.* 2003). The effects β are the *q*-dimensional effect of the covariate *c* (*i.e.*, log of the centroid size, gender and direction-of-cross) or of the genotype *j* representing the direction and magnitude of the shape change of the overall configuration of landmarks within the shape space.

A forward/backward algorithm was used for multiple QTL model searching. This procedure drops and refines positions of additive QTL without any prior knowledge of their number per chromosome, and compares models based on a penalized LOD score (Broman and Speed 2002; Broman and Sen 2009; Manichaikul *et al.* 2009), $pLOD\left(\text{γ}\right)=-\log_{10}p-T\left|\text{γ}\right|$. The forward search was repeated up to a model γ including 50 QTL. The penalty *T* for each additional QTL was evaluated using the 1000 permutations approach (Churchill and Doerge 1994). One classic inferential approach in geometric morphometrics uses the sum of the residual sum of squares (Goodall 1991). Here, pLOD scores are derived from the Pillai’s trace, a classic multivariate statistic, which makes use of covariances and is proven to be fairly robust in a variety of situations (Olson 1974, 1976; Tabachnick and Fidell 2013). However, it is important to note that recent approaches to decipher the basis of adaptation or speciation using RAD-seq or similar techniques (see Jones and Good 2016, for a review) in nonmodel organisms may have fairly small sample sizes compared to the dimensionality of phenotypes (*e.g.*, Huber *et al.* 2015). Such n≪p studies might gain in robustness by focusing just on variances, dropping the cost of estimating covariances, as suggested also in other contexts (Adams 2014). Our multivariate approach for mapping multiple QTL is very similar to the one developed for the mapping of function-valued traits, with pLOD based on the sum of the residual sum of squares (Kwak *et al.* 2014; Gray *et al.* 2015). Bayes credible intervals of QTL were computed from the $10^{\text{LOD}\left(\theta \right)}$ profile (Dupuis and Siegmund 1999; Sen and Churchill 2001; Manichaikul *et al.* 2006).

The magnitude of shape changes associated with each QTL was expressed first in units of Procrustes distance as the norm of the additive vector $\left\|\beta_{j}\right\|={\left(\beta_{j}\beta_{j}^{t}\right)}^{0.5}$ (Klingenberg *et al.* 2001; Workman *et al.* 2002). Also, the amount of variation accounted for, given all the other QTL and covariates, was reported as the percentage of total Procrustes variance (%SST in Supplemental Material, Table S1 and Table S2), a statistic routinely used with multivariate linear models (*e.g.*, Monteiro 1999). We also reported the effect size as a percentage of variance accounted for in the specific direction defined by the additive vector $\beta_{j}$ in the shape space (%SS proj Scores in Table S1 and Table S2). For that, we defined a new shape variable, $v=Y\beta_{j}^{t}{\left(\beta_{j}\beta_{j}^{t}\right)}^{-0.5}$, corresponding to the shape variable most associated with the shape changes defined by $\beta_{j}$ and containing both the effect and the residuals in that specific direction (Drake and Klingenberg 2008). The proportion of those projection scores explained by the QTL *j* is then the ratio of variance between the $E\left(v|p_{j}\right)$ and the scores **v**. Its expectation with backcross and unlinked QTL is $h_{j}^{2}=\frac{\sum_{k}\beta_{j,k}^{2}}{\beta_{j}\left(B^{t}B+4\times {\text{Σ}}_{e}\right)\beta_{j}^{t}{\left(\beta_{j}\beta_{j}^{t}\right)}^{-1}}$.

QTL-based **G** matrices were compared across the three approaches using only the xy(z)-coordinates of the 13 manual landmarks that were common across the set of matrices to be compared. Many approaches for matrix similarity have been used in the literature for comparing **G** matrices across populations (*e.g.*, Aguirre *et al.* 2013; Teplitsky *et al.* 2014) or between levels of variation (see for example Debat *et al.* 2009, for contrasting canalization and developmental stability). Overall distance between **G** matrices was assessed with the root Euclidean distance (Dryden *et al.* 2009), $d_{H}\left(G_{1},G_{2}\right)=\left\|G_{1}^{1/2}-G_{2}^{1/2}\right\|$ with the matrix square root $G^{1/2}=U\Lambda^{1/2}U^{-1}$ where $U\Lambda U^{-1}$ is the spectral decomposition of **G**. Then, the similarity between $g_{\text{max}}$ was measured as their angle, which was compared to 100,000 pairs of random vectors to assess the significance of whether two $g_{\text{max}}$ were more similar than pairs of random vectors (Klingenberg and Leamy 2001). To extend this comparison beyond $g_{\text{max}}$ and pairwise comparisons, we computed the common subspace **H** across the three **G** (Krzanowski 1979). The matrix $H=\sum_{i=1}^{3}A_{i}A_{i}^{t}$, where $A_{i}$ corresponds to the first $q_{i}$ eigenvectors of $G_{i}$. We chose $q_{i}$ based on the cumulative amount of variance accounted for (≥90%). Briefly, the spectral decomposition of **H** provided axes maximizing the similarity among matrices $\Delta =\sum_{i=1}^{3}{\text{cos}}^{2}\delta_{i}$, where $\delta_{i}$ is the angle between an eigenvector and the subspace $A_{i}$. An upper bound of Δ is the number of matrices to compare. Angles $\delta_{i}$ quantified how different each eigenvector of **H** are from the $q_{i}$ eigenvector of $G_{i}$ (*e.g.*, see Aguirre *et al.* 2013 for a more detailed treatment). Finally, we compared the three datasets based on their difference in terms of heritability. With multidimensional traits, heritability is also multidimensional (Klingenberg 2010), meaning that beyond an overall amount of heritable variance there are also directions across the shape space accounting for a varying degree of heritable variation. The multivariate analog to $h^{2}$ is $GP^{-}$, where ^−^ stands for the Moore-Penrose generalized inverse. Its spectral decomposition $U\Lambda U^{-1}$ provided these directions in the shape space maximizing heritability (Klingenberg and Leamy 2001).

These shape features $u_{h}$ as well as any eigenvector of **G**, **P**, or QTL effects $\beta_{j}$ might then be amplified and visualized as a colorized 3D surface using thin-plate spline mapping from the mean shape and its mesh model, and by computing the signed distance between these extrapolated meshes. This visualization procedure made use of tps3d and meshDist functions from the R/Morpho package (Schlager 2015a).

### Comparison with previous mapping data

Mandible shape QTL have already been assessed in several studies using 2D landmarks on F2 mice from a LG/J×SM/J intercross (Klingenberg *et al.* 2001, 2004) or on F3 mice from the same cross (Leamy *et al.* 2008). Despite missing confidence intervals for the discovered QTL, the latter was kept for comparison, because it represented a follow-up study on the former two with more markers and more individuals. A genome-wide association study using the first generation of wild-caught mice from a hybrid zone (Pallares *et al.* 2014) was also included in comparisons. It differs from the three others by its use of outbred hybrid mice, 3D data, but smaller sample size (Table 1).

**Table 1:** Study design and descriptive statistics for confidence interval on QTL positions

Study*^a^*	N Land	Dim	N Mrk	N Ind	Age*^b^*	Cross	N QTL	Q1*^c^*	Median	Q3	∑
Present-2D	13	2D	822	427	28	N2:(B6×AJ)×AJ	17	6	9	20	276.8
Present-3D	13	3D	882	427	28	N2:(B6×AJ)×AJ	19	7	9	17.3	271.2
Present-3D Semiland	13 + 579	3D	882	427	28	N2:(B6×AJ)×AJ	23	2.5	5.1	9.2	168.9
Klingenberg *et al.* (2001)	5	2D	76	476	70	F2:LG×SM	23	12	16.9	29.6	477
Klingenberg *et al.* (2004)	16	2D	76/96	954	70	F2:LG×SM	32	8.5	13.3	24	560
Leamy *et al.* (2008)	15	2D	353	374 + 1515	70	F2+F3:LG×SM	37				
Pallares *et al.* (2014)	14	3D	145,378	178	63-84	F1:wild-caught	10	0.04	0.09	0.12	0.9

N Land, ; Dim, ; N Land, number of landmarks; Dim, dimensionality; N Mrk, number of markers; N Ind, number of individuals; N QTL, number of quantitative trait loci; Q1, first quartile of the confidence intervals; Q3, third quartile of the confidence intervals; Σ, sum of the confidence intervals etc.

a For the Klingenberg studies, QTL with markers now known to be syntenic were either removed or replaced with the central position of the QTL when possible. See text for further details.

b Age in d.

c Intervals are given in cM according to the current genetic map (Cox *et al.* 2009) and were converted from earlier map or physical position using the Jackson Laboratory’s Marker Query Tool.

The closest proximal and distal markers given for the confidence intervals in earlier studies were converted to the current genetic map (Cox *et al.* 2009) using the Jackson Laboratory’s Marker Query Tool. The SNPs used in the mapping from F3 of the LG/J×SM/J intercross (Leamy *et al.* 2008) were converted to the Cox map using the Mouse Map Converter tool of the Jackson Laboratory using the SNP IDs. Six SNPs were not recovered from their IDs in the conversion. Genomic positions in the NCBI build37 for these SNPs were known from the updated heterogeneous stock data (Shifman *et al.* 2006; Valdar *et al.* 2006). They were then converted from those NCBI build37 genomic coordinates to the Cox map using the Mouse Map Converter tool. The genomic positions of loci discovered in Pallares *et al.* (2014) were converted from the GRCm38 coordinates to the Cox map using the same tool.

Some markers used in LG × SM studies (Klingenberg *et al.* 2001, 2004) are now known to be syntenic and do not localize to a specific location in the genome. Overall, three QTL were removed from the comparison, and left or right positions of the confidence interval were imputed with values from the central position for three others. The central positions of two other QTL were treated as missing because the new map positions fell outside the confidence interval, and the closest flanking marker was several dozen cM away. All these imputations led to slightly underestimated descriptive statistics on confidence intervals from previous studies. Such bias is nonetheless against any evidence of gain in the precision of QTL locations with more recent data.

### Data availability

Genotypes and phenotypes are available from File S1 as a cross object readable by R/qtl or R/shapeQTL.

## Results

### Mandible shape variation

**Figure 2:**
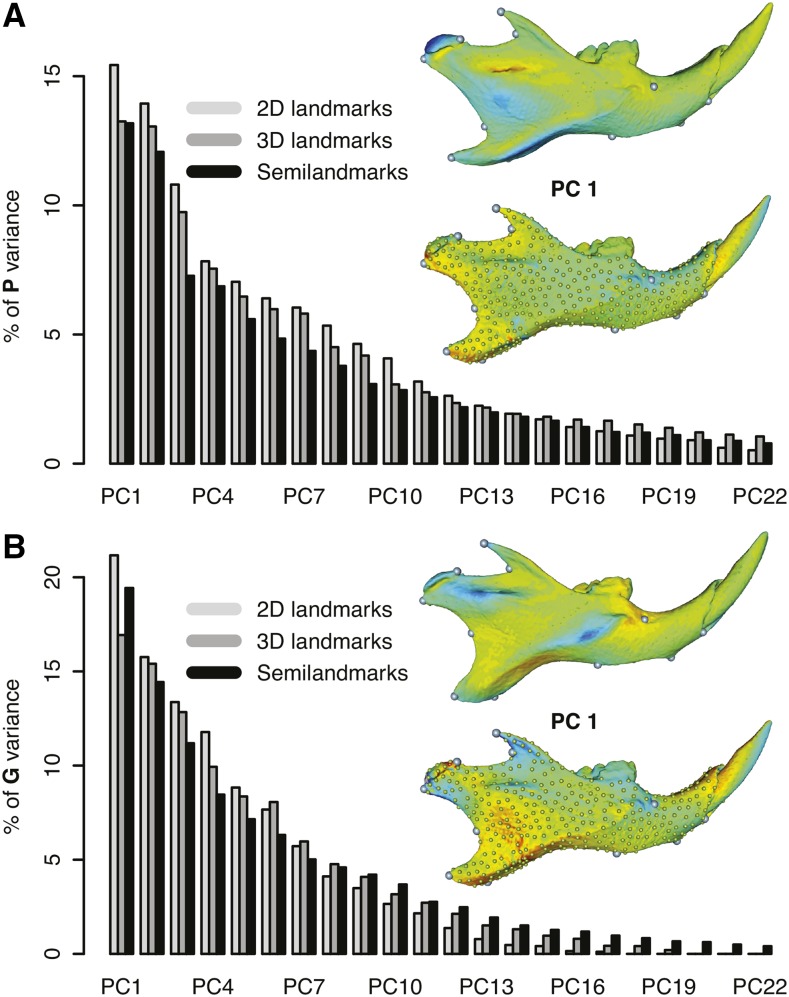
Principal component (PC) analyses of mandible shape variation. (A) Percentage of variance explained by the first 22 PCs from phenotypic covariance matrices together with the visualization of shape changes associated with PC 1 from 3D landmark and semilandmark datasets. In the visualization, darker colors (red or blue) represent interpolated shape changes diverging from the mean shape. Green color represents unchanged shape of the mandible compared to the mean shape. (B) Percentage of variance explained by the first 22 PCs from **G** matrices based on discovered QTL (quantitative trait loci).

Variation from expert-annotated 3D landmarks was dispersed with 22 out of 32 principal components (PC) with a variance higher than 1%, and explaining 94% of the total Procrustes variance. Two first PCs explained about 13% each (Figure 2A). Interestingly, the interpolated shape changes (using thin-plate-spline) associated with PC1 show strong correlated deformations in muscle insertion regions where no landmarks are digitized to actually capture genuine variation in these regions. Shape variation in 2D was dispersed with 18 out of 22 PCs with a variance higher than 1%, explaining 97% of the total Procrustes variance and the first three PCs explaining between 10–14% each. Based only on the xy-coordinates of the 13 landmarks, major axes of variation are more similar than two random vectors ($\alpha =25.3{}^{\circ},p<10^{-5}$) once the permutation between PC1 and PC2 on 3D landmarks, which account for almost the same amount of variance, is controlled.

Semilandmark variation was most dispersed with 426 nonnull eigenvalues from which 19 PCs, with a percentage of explained variance higher than 1%, explained 79% of the total Procrustes variance, the first two explaining 13% and 14% each. We chose to use the first 71 PCs that accounted for 95% of the total variance. Based only on fixed landmarks, the main axis of phenotypic variation is very similar to PC1 from 2D landmarks ($\alpha =40.9{}^{\circ},p=2\times 10^{-5}$), or to PC2 from the 3D landmarks ($\alpha =26.6{}^{\circ},p<10^{-5}$).

### Covariate analysis

The main effects of centroid size, gender, and directionality of the cross on mandible shape were found to be significant (*p* < 0.0001) but no significant interactions among them were found. Altogether, they explained 4.5% of the total Procrustes variance. The direction-of-cross was the major effect in this sample, explaining 2.5% of the total Procrustes variance (Table S1). Covariate results with 2D shapes were similar to the 3D data (Table S1). Results from the multivariate linear model of the three covariates were similar to those from the 3D manual landmark only, with the covariates explaining 5.5% of the variance altogether.

### 2D embedding of shape variation

**Figure 3:**
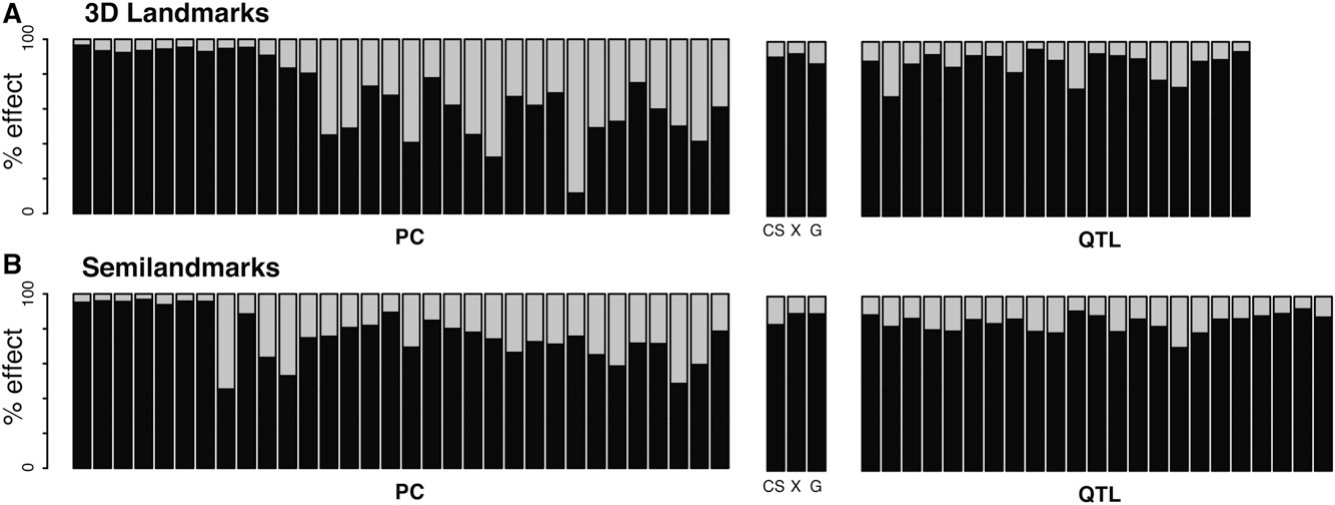
Percentage of shape changes within the xy-plane (black) and along the *z* direction (gray). The proportion of shape effects *β* that lie along the $m^{th}$ dimension (*m*= 1, 2, or 3) is the sum of the squared effects over the *k* landmarks on the $m^{th}$ dimension normalized by the norm of the effect, $\sum_{i=1}^{k}\beta_{i,m}^{2}/\left\|\beta \right\|$. The figure may be understood as the proportion of changes that are embedded in the flat plane (xy) or that get out of this plane (*z*). Such parametrization is sensical here, despite the fact that shapes are invariant to rotation by definition, only because they were specifically oriented according to this specific coordinate system. (A) Principal components (PC), covariate, and QTL (quantitative trait loci) effects for 3D landmarks. (B) Principal components, covariate, and QTL effects for semilandmarks. Covariates are noted CS for the log of the centroid size, X for the direction of the cross, and G for gender.

3D shapes from manual landmarks were oriented according to the reference 2D plane allowing the decomposition of each effect according to the antero-posterior and bucco-lingual axes (Figure 3). The variation of the 3D landmarks on each PC in the bucco-lingual direction is highly variable (light gray amount in Figure 3). However, major axes of variation (PC1 to PC10; ∼73.6% of the total Procrustes variance) are embedded mainly in the antero-posterior/dorso-ventral plane of the mandible (black and dark gray), with very little of this variation in the bucco-lingual direction (4.4%). Accordingly, only 9–13% of covariate effects (size, sex, or direction-of-cross) were along this bucco-lingual axis. Shape variation from the semilandmarks was again mostly embedded in the flat plane with only 5.9% of the variation in the bucco-lingual direction, and covariate effects were mostly within this plane (∼90% of the effect).

### QTL mapping of mandible shape

**Figure 4:**
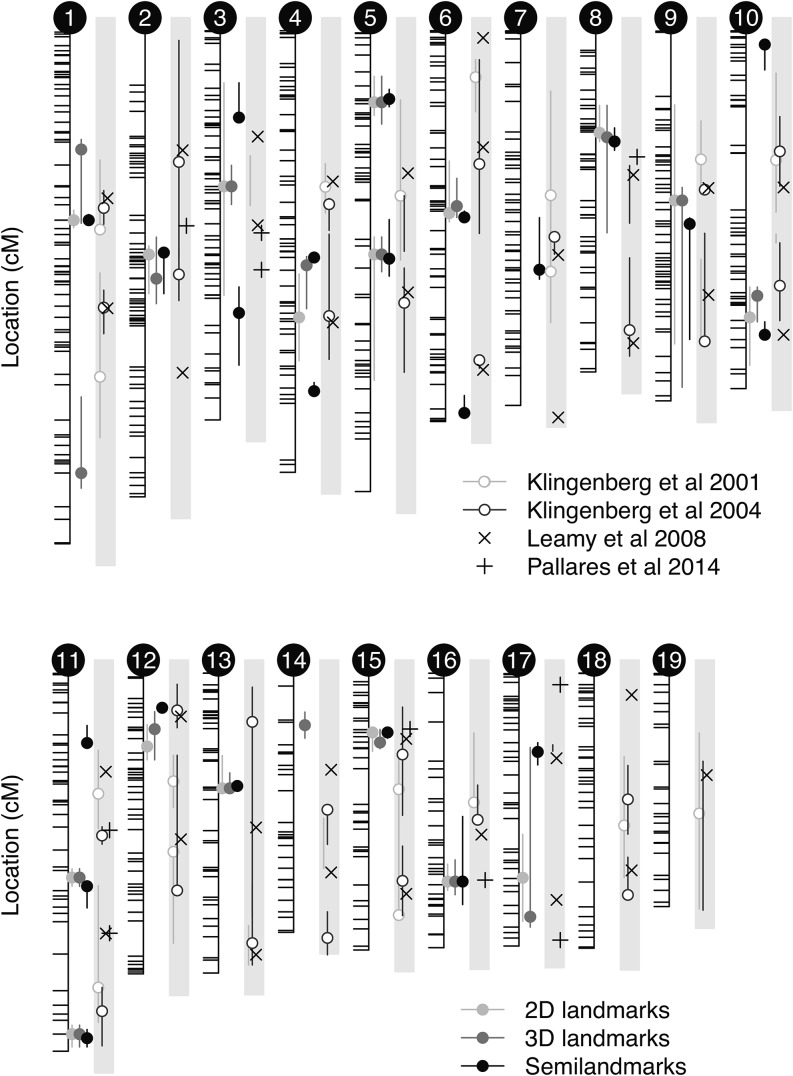
QTL from 2D, 3D, and semilandmark analyses. Results from earlier studies from the LG/J×SM/J intercrosses (Klingenberg *et al.* 2001, 2004; Leamy *et al.* 2008) and from the Pallares *et al.* (2014) GWAS (genome-wide association study) are plotted in the gray boxes.

In all three cases (2D, Manual 3D landmarks, and Semilandmarks), the three covariates (log of the centroid size, gender, and direction-of-cross) were included in the QTL mapping as additive covariates. Multivariate QTL mapping identified between 17 QTL for 2D landmarks, 19 QTL for 3D manual landmarks, and 23 QTL for the 3D semilandmark analysis. All autosomes but two (chromosomes 18 and 19) harbor at least one, and in some cases up to three, QTL (chromosome 11) depending on the phenotyping method used. Summaries of the positions of the discovered QTL (location, nearest marker, and the confidence intervals) are provided in Table 2 for semilandmarks. Summaries for all three cases are provided in Table S2 and plotted on Figure 4. The median widths of the confidence intervals were 9 cM for both 3D landmarks and their 2D projections, and 5 cM for the semilandmark dataset, and three quarters of the confidence intervals were smaller than 17.3 cM, 20 cM, and 9.2 cM, respectively, for these three datasets (Table 1). Thirteen QTL were replicated across the three approaches, all 2D QTL were replicated in 3D but some were split in two with the semilandmark data or were not captured, eight were only mapped with the semilandmarks, and two only with the 3D landmarks (Figure 4 and Table S2).

**Table 2:** Closest SNP, confidence interval, and protein-coding gene content of semilandmark QTL

QTL	Closest SNP	Chr	Left	Pos	Right	Replic.	nPCG*^a^*	nCG.2D*^b^*	nCG.3D	CG.Semi*^c^*
SH1	gnf01.075.385	1	42.33	43.62	44.33	2D, 3D	56		4	
SH2	rs3722345	2	51.09	51.09	60.54	2D, 3D	243	2	2	
SH3	rs6274061	3	12.01	20.01	21.01		81	2		
SH4	rs3676039	3	59.01	65.01	77.01		115	2		*Lef1*
SH5	rs3711477	4	52.01	52.20	53.01	3D	12	1		
SH6	UT_4_132.137715	4	81.01	83.01	84.01		44			***Rere***
SH7	rs13478154	5	13.50	15.71	17.50	2D, 3D	75	6	9	*Shh*, *Drc1*, *Ift172*
SH8	rs13478388	5	43.50	52.50	56.50	2D, 3D	345	12	6	*Ambn*, ***Fras1***, *Prkg2*, *Dmp1*, ***Idua***, *Fgfrl1*, ***Mn1***, *Kctd10*
SH9	CEL-6_86289708	6	41.55	43.00	43.00	2D, 3D	3	4	3	
SH10	rs3658783	6	84.00	88.00	89.28					
SH11	rs13479427	7	43.05	55.02	57.20		477			***Akap13***, ***Kif7***, *Serpinh1*, *Folr1*
SH12	rs6386110	8	22.38	25.52	27.52	2D, 3D	69	1	7	
SH13	rs3721056	9	43.10	44.47	71.10	2D, 3D	366	2	2	***Atr***, *Ryk*
SH14	rs3686911	10	3.03	3.18	9.03		48			
SH15	mCV24217147	10	67.03	70.03	71.12	2D, 3D	24	3	1	
SH16	rs3700830	11	12.08	16.08	17.08		52			
SH17	rs13481127	11	48.08	49.08	54.08	2D, 3D	116			
SH18	rs3672597	11	82.08	84.08	86.08	2D, 3D	106			
SH19	rs13481321	12	6.90	7.99	8.95	3D	44			
SH20	rs3693942	13	25.00	26.00	26.52	2D, 3D	36			
SH21	CEL-15_36490596	15	13.68	13.68	14.99	2D, 3D	32			
SH22	rs4204106	16	33.03	48.03	53.31	2D, 3D	177			
SH23	rs6298471	17	16.03	18.14	21.14		314			

QTL, quantitative trait loci; Chr, chromosome; Pos, position; Replic, replication, nPCG, number of protein coding genes; nCG, number of candidate genes; SH, shape etc.

a Number of protein-coding genes in the interval.

b Number of candidate genes annotated for “mandible” in the MGI databases in the QTL confidence interval from 2D or 3D datasets.

c Candidate genes annotated for “mandible” in the MGI databases in the QTL confidence interval for the semilandmark analysis. Candidates with nonsynonymous or splice-site variants between AJ and C57BL/6J are indicated in bold.

Effect sizes of QTL were small (∼1% of total Procrustes variance; Table S2) but explained on average ∼12% of the variance in the specific direction defined by the QTL effect (regression projection scores). Replicated QTL between 2D and 3D data explained a similar percentage of total Procrustes variance (Wilcoxon test: $V_{16}=57,p=0.82$), but 3D effects explained a significantly greater percentage of variance in the specific direction of the QTL (projection scores) than 2D effects (Wilcoxon test: $V_{16}=153,p=7.63\times 10^{-6}$). According to shape variation and covariate effects, QTL act mainly in the 2D plane (Figure 3). However, the contribution of the third dimension may be as high as 30% for some QTL with the manual landmarks or the semilandmarks (Figure 3). It is important to note that these QTL were not detected in the 2D analysis (Table S2). Visualization of QTL effects from 3D landmarks only or semilandmarks are provided in supplementary Figure S1 and Figure S2.

### Comparison of QTL-based G matrices

Altogether, these QTL accounted for 14.6% (2D), 15.1% (3D), and 16.9% (semilandmarks) of the total Procrustes variance. From those, 12.8% (3D) and 14.4% (semilandmarks) of the genetic variance are along the third dimension. Overall differences among the three $G_{2k}$ matrices measured by the root Euclidean distances $d_{H}$ are low, the semilandmark matrix appearing as the most different ($d_{\text{Semi}-2\text{D}/3\text{D}}=0.012$ and $d_{2\text{D}-3\text{D}}=0.002$). In agreement with this observation and similarly to **P** matrices, **G** matrices presented a very similar eigenvalue profile among the three approaches (Figure 2) with $g_{\text{max}}$ explaining about 20% of the variance. Here, there was also a permutation between the two first PC in the 3D landmarks compared to the two other datasets. Once this permutation was controlled, these three $g_{\text{max}}$ were more similar than random vectors $\left(\alpha_{2\text{D}/3\text{D}}=18.2{}^{\circ},p<10^{-5},\alpha_{2\text{D}/\text{Semi}}=61.4{}^{\circ},p=0.012,\alpha_{3\text{D}/\text{Semi}}=69{}^{\circ},p=0.025\right)$ based only on the xy-coordinates of the 13 fixed landmarks for the two first comparisons and on xyz-coordinates for the last one.

The Krzanowski’s common subspace analysis confirmed these observations. Angles between the first seven eigenvectors of the common subspace **H** and the *q* PCs accounting for 90% of the variance for each matrices (q=9 for 2D landmarks or 10 for the $G_{2k}$ based on 3D landmarks or semilandmark datasets) were small (δ ranging from 1.6–16°). Their associated eigenvalues were very close to their maximum value of three (Δ ranging from 2.997–2.859). This means that the common subspace may be almost perfectly recovered from linear combinations of the $q_{i}$ eigenvectors of any of the $G_{2k}$ matrices. The semilandmark $G_{2k}$ matrix appeared again to be the quickest to diverge, underlying the additional information we get from semilandmarks even if they were not explicitly taken into account in the construction of the common subspace.

**Figure 5:**
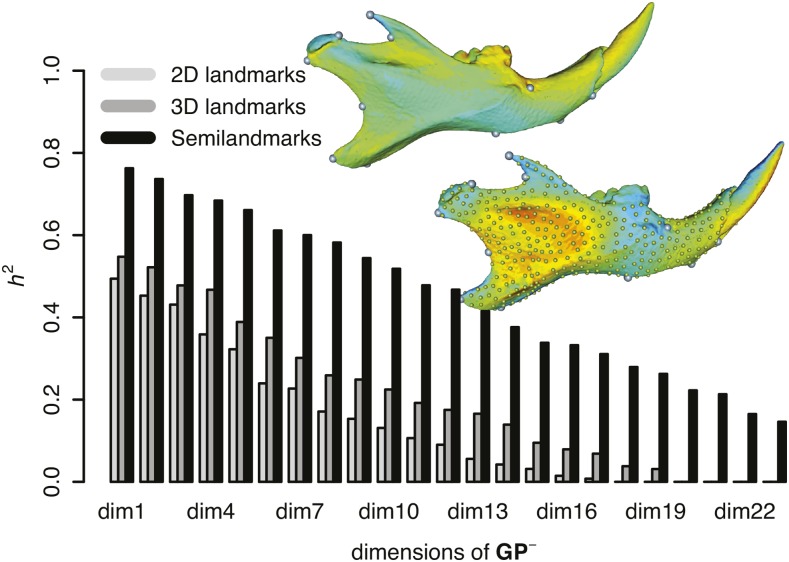
Multivariate heritabilities of shape dimensions. The $h^{2}$ are the eigenvalues of the $GP^{-}$ matrix. The shapes correspond to the shape changes associated with the first dimension of $GP^{-}$ for the 3D landmark or the semilandmarks. For the significance of the color see Figure 2.

Multivariate heritabilities are systematically greater with 3D than 2D data (Figure 5) but in the same order ranging from 0.55–0.01. Semilandmarks show dimensions with a heritability ranging from 0.76–0.15. The shape changes associated with the most heritable dimension were very different to the one observed on 3D landmarks only (Figure 5). Such discrepancies seem easily explained as the strong changes appeared to map on mandible surface and ridges where no manual landmarks could be easily captured.

## Discussion

Previous studies using QTL mapping of mandible size and shape in mouse have relied typically on 2D landmarks and sparse sampling of the genome using microsatellite markers (Klingenberg *et al.* 2001, 2004) or a few hundred SNPs (Leamy *et al.* 2008). Our primary purpose in this study was to validate QTL responsible for the variation in mandibular shape observed in mice using 3D phenotyping with denser genotyping than previously attempted, and assess the gains (if any) from the consideration of the third dimension.

We detected marginally fewer QTL than studies based on the LG/J×SM/J intercross (Table 1). This might be expected as we fitted a model with all QTL simultaneously, whereas the LG/J×SM/J intercross studies modeled the genetics of the trait at the chromosome-wide level and used sample sizes about three times bigger. Effects of sample size and/or of allele frequencies seem striking in the study of Pallares *et al.* (2014), where only 10 loci were discovered but with greater precision, thanks to their use of an outbred population and a dense SNP map. Overall, about half of the 23 QTL we discovered had already been reported in the literature. Only one region on chromosome 15 seemed to be replicated across the three different crosses (Figure 4). Another GWAS locus on chromosome 16 was replicated in our cross. Two QTL previously detected with imprinting due to maternal genetic effects (Leamy *et al.* 2008) were also replicated here. At the time of the submission, a 3D geometric morphometric study using about 700 laboratory outbred mice, 80,000 SNPs, and univariate association mapping of the main principal components was published (Pallares *et al.* 2015). They mapped seven loci for mandible shape with great precision and their main finding, which was related to the *Mn1* gene, seems to be replicated here based on gene content (SH8 on chromosome 5 in Table 2).

It may initially appear that not much difference exists between the mapping results based on 3D manual landmarks and their 2D projections, due to their significant overlap (Figure 4). However, the benefit of adding the third dimension is demonstrated by the reduction in the confidence intervals of the QTL estimates. The median CI estimate for our 2D projection results was 11 cM. Yet, when we conducted the mapping on 3D landmarks, extreme CIs were marginally reduced and median CI was down to 6.3 cM when we used semilandmarks. The difference was even more striking when we considered the total length of the QTL intervals, which dropped by 120 cM, representing a 38% drop in the number of known protein coding genes with 75% of QTL covering at most 146 protein coding genes (358 with 2D and 271 with 3D landmarks). The 2D projection data yielded very similar results to the one of 13.3 cM obtained from the remaining 24 QTL reported in earlier studies (Klingenberg *et al.* 2001, 2004) despite a marked difference in molecular marker density (10-fold).

The consideration of the third dimension leads to the discovery, with 3D landmarks, of two additional QTL on chromosome 1 and 14, both QTL covering a few mandible-annotated genes (*Disp1*, *Hhat*, *Ifr6*, and *Kat6b*). Adding one dimension to each landmark (*i.e.*, 10 independent dimensions to the shape space) increases detection power. This increase in power is not just related to bigger effect sizes or higher dimensionality, which could be more costly than useful in some cases (Healy 1969; Adams 2014). The two additional QTL actually have 26% and 32% of their effects along these additional dimensions. Thus, the specific consideration of the third dimension in the mapping is clearly very informative in these cases. The relaxed constraints on homology of semilandmarks and their ability to densely model the surface of bone led to the discovery of eight additional QTL. Three of them show between 20–30% of their effect on the third dimension. Three of those additional QTL cover one to four mandible-annotated candidate genes: *Lef1*, *Rere*, and in the same QTL *Akap13*, *Kif7*, *Serpinh1*, and *Folr1*. The overall additive genetic variation (**G**) is consistent between 2D and 3D data, both in terms of the amount of total variation captured and the directionality of major variations. Beyond an overall similarity with other data, semilandmarks capture original shape features related to the bone surface, and those features drive the pattern of multivariate heritability ($GP^{-}$).

Multivariate approaches have been repeatedly shown to be more powerful than analyzing individual PCs (or univariate traits) independently in various contexts (see for instance in GWAS, Galesloot *et al.* 2014; Gao *et al.* 2014; Stephens 2013). However, this univariate PC approach is commonly used, at least for operational reasons (*e.g.*, Pallares *et al.* 2014, 2015; Percival *et al.* 2016, with geometric morphometric data). An incidental result of our study is a plea for the use of a fully multivariate approach with shape data, not only because shape is a single multidimensional trait (Klingenberg and Gidaszewski 2010; Collyer *et al.* 2015). Major phenotypic PCs appear to have only minor components of variance on the third dimension, whereas some QTL present up to a third of their variation in that specific direction. Prior selection of shape variables based on phenotypic PCA will reduce effect sizes by a third for those QTL, thus reducing power. We have chosen to reduce the dimensionality of the semilandmark data using such a technique. However, we kept most of the total variance (95%) while discarding a large amount of nonnull dimensions (355). Our assumption is that the remaining variance was only random variation. Such noisiness is inherent to the registration process of semilandmarks. This assumption leads us to not reestimate the effects and their associated effect sizes on the complete shape, as doing so will only marginally change our estimates. However, this reestimation of QTL effects on the complete shape may be good practice when the QTL detection is, for some reason, done on a strongly reduced shape space (Pallares *et al.* 2014, 2015; Liu *et al.* 2014; Zeng *et al.* 2000; Langlade *et al.* 2005; Mezey *et al.* 2005; Franchini *et al.* 2013). Major QTL may be detected with the first PCs only, but they also have pleiotropic effects on additional dimensions.

In conclusion, the congruence of our results pleads for robustness of our knowledge on the genetic architecture of the mouse mandible, built over a few decades and initially based on 2D imaging techniques. There are many benefits of performing 2D morphometrics in large phenotyping programs, which can be summarized as the simplicity of the techniques and the time saved, but they come at the price of accuracy. Despite inherent difficulties and workloads that may impede the broader use of 3D techniques, information can be gained even from fairly flat structures like the mouse mandible. Once these technical and cost difficulties have been overcome, it appears that making the most of new technologies by opting for denser phenotyping is worth the supplementary technical expertise that is required, and may provide some new insights on the genetics of shape.

## Supplementary Material

Supporting Information

## References

[bib1] Adams D. C., 2014 A method for assessing phylogenetic least squares models for shape and other high-dimensional multivariate data. Evolution 68: 2675–2688. 24899536 10.1111/evo.12463

[bib2] Aguirre J. D. Hine E. McGuigan K. Blows M. W., 2013 Comparing G: multivariate analysis of genetic variation in multiple populations. Heredity 112: 21–29. 23486079 10.1038/hdy.2013.12 PMC3860158

[bib3] Anderson P. S. Renaud S. Rayfield E. J., 2014 Adaptive plasticity in the mouse mandible. BMC Evol. Biol. 14: 85. 24742055 10.1186/1471-2148-14-85 PMC4002541

[bib4] Andolfatto P. Davison D. Erezyilmaz D. Hu T. T. Mast J., 2011 Multiplexed shotgun genotyping for rapid and efficient genetic mapping. Genome Res. 21: 610–617. 21233398 10.1101/gr.115402.110 PMC3065708

[bib5] Aneja D. Vora S. R. Camci E. D. Shapiro L. G. Cox T. C., 2015 Automated Detection of 3D Landmarks for the Elimination of Non-Biological Variation in Geometric Morphometric Analyses. 28th IEEE International Symposium on Computer-Based Medical Systems (22–25 June São Carlos, Brazil). 10.1109/CBMS.2015.86 PMC4526271 26258171

[bib6] Atchley W. R. Hall B. K., 1991 A model for development and evolution of complex morphological structures. Biol. Rev. Camb. Philos. Soc. 66: 101–157. 1863686 10.1111/j.1469-185x.1991.tb01138.x

[bib7] Atchley W. R. Plummer A. A. Riska B., 1985 Genetics of mandible form in the mouse. Genetics 111: 555–577. 4054609 10.1093/genetics/111.3.555 PMC1202658

[bib8] Baird N. A. Etter P. D. Atwood T. S. Currey M. C. Shiver A. L., 2008 Rapid SNP Discovery and Genetic Mapping Using Sequenced RAD Markers. PLoS One 3: e3376–e3377. 18852878 10.1371/journal.pone.0003376 PMC2557064

[bib9] Boell L., 2013 Lines of least resistance and genetic architecture of house mouse (*Mus musculus*) mandible shape. Evol. Dev. 15: 197–204. 23607303 10.1111/ede.12033

[bib10] Boell L. Tautz D., 2011 Micro-evolutionary divergence patterns of mandible shapes in wild house mouse (*Mus musculus*) populations. BMC Evol. Biol. 11: 306. 22008647 10.1186/1471-2148-11-306 PMC3213108

[bib11] Boell L. Gregorova S. Forejt J. Tautz D., 2011 A comparative assessment of mandible shape in a consomic strain panel of the house mouse (*Mus musculus*)–implications for epistasis and evolvability of quantitative traits. BMC Evol. Biol. 11: 309. 22011306 10.1186/1471-2148-11-309 PMC3212827

[bib12] Boell L. Pallares L. F. Brodski C. Chen Y. Christian J. L., 2013 Exploring the effects of gene dosage on mandible shape in mice as a model for studying the genetic basis of natural variation. Dev. Genes Evol. 223: 279–287. 23563729 10.1007/s00427-013-0443-y PMC4013528

[bib13] Bookstein F. L., 1997 Landmark methods for forms without landmarks: morphometrics of group differences in outline shape. Med. Image Anal. 1: 225–243. 9873908 10.1016/s1361-8415(97)85012-8

[bib14] Broman, K. W., and S. Sen, 2009 *A Guide to QTL Mapping with R/qtl*, Springer, New York.

[bib15] Broman K. W. Speed T. P., 2002 A model selection approach for the identification of quantitative trait loci in experimental crosses. J. R. Stat. Soc. Series B Stat. Methodol. 64: 641–656.

[bib16] Broman K. W. Wu H. Sen S. Churchill G. A., 2003 R/qtl: QTL mapping in experimental crosses. Bioinformatics 19: 889–890. 12724300 10.1093/bioinformatics/btg112

[bib17] Bromiley P. A. Schunke A. C. Ragheb H. Thacker N. A. Tautz D., 2014 Semi-automatic landmark point annotation for geometric morphometrics. Front. Zool. 11: 61.

[bib18] Cande J. Andolfatto P. Prud’homme B. Stern D. L. Gompel N., 2012 Evolution of multiple additive loci caused divergence between *Drosophila yakuba* and *D. santomea* in wing rowing during male courtship. PLoS One 7: e43888. 22952802 10.1371/journal.pone.0043888 PMC3431401

[bib19] Cardini A., 2014 Missing the third dimension in geometric morphometrics: how to assess if 2D images really are a good proxy for 3D structures? Hystrix 25: 73–81.

[bib20] Chevalier F. Valentim C. L. LoVerde P. T. Anderson T. J., 2014 Efficient linkage mapping using exome capture and extreme QTL in schistosome parasites. BMC Genomics 15: 617. 25048426 10.1186/1471-2164-15-617 PMC4117968

[bib21] Churchill G. A. Doerge R. W., 1994 Empirical threshold values for quantitative trait mapping. Genetics 138: 963–971. 7851788 10.1093/genetics/138.3.963 PMC1206241

[bib22] Collyer M. L. Sekora D. J. Adams D. C., 2015 A method for analysis of phenotypic change for phenotypes described by high-dimensional data. Heredity 115: 357–365. 25204302 10.1038/hdy.2014.75 PMC4815463

[bib23] Cox A. Ackert-Bicknell C. L. Dumont B. L. Ding Y. Bell J. T., 2009 A new standard genetic map for the laboratory mouse. Genetics 182: 1335–1344. 19535546 10.1534/genetics.109.105486 PMC2728870

[bib24] Debat V. Debelle A. Dworkin I., 2009 Plasticity, canalization, and developmental stability of the Drosophila wing: joint effects of mutations and developmental temperature. Evolution 63: 2864–2876. 19624729 10.1111/j.1558-5646.2009.00774.x

[bib25] Drake A. G. Klingenberg C. P., 2008 The pace of morphological change: historical transformation of skull shape in St Bernard dogs. Proc. Biol. Sci. 275: 71–76. 17956847 10.1098/rspb.2007.1169 PMC2562403

[bib26] Dryden, I. L., and K. V. Mardia, 1998 *Statistical Shape Analysis*, Wiley, Hoboken, New Jersey.

[bib27] Dryden I. L. Koloydenko A. Zhou D., 2009 Non-Euclidean statistics for covariance matrices, with applications to diffusion tensor imaging. Ann. Appl. Stat. 3: 1102–1123.

[bib28] Dupuis J. Siegmund D., 1999 Statistical methods for mapping quantitative trait loci from a dense set of markers. Genetics 151: 373–386. 9872974 10.1093/genetics/151.1.373 PMC1460471

[bib29] Dworkin I. Anderson J. A. Idaghdour Y. Parker E. K. Stone E. A., 2011 The effects of weak genetic perturbations on the transcriptome of the wing imaginal disc and its association with wing shape in Drosophila melanogaster. Genetics 187: 1171–1184. 21288875 10.1534/genetics.110.125922 PMC3070525

[bib30] Elshire R. J. Glaubitz J. C. Sun Q. Poland J. A. Kawamoto K., 2011 A robust, simple genotyping-by-sequencing (GBS) approach for high diversity species. PLoS One 6: e19379. 21573248 10.1371/journal.pone.0019379 PMC3087801

[bib31] Emília Santos M. Berger C. S. Refki P. N. Khila A., 2015 Integrating evo-devo with ecology for a better understanding of phenotypic evolution. Brief. Funct. Genomics 2015: 1–12. 10.1093/bfgp/elv003 PMC4652033 25750411

[bib32] Fedorov A. Beichel R. Kalpathy-Cramer J. Finet J. Fillion-Robin J.-C., 2012 3D Slicer as an image computing platform for the quantitative imaging network. Magn. Reson. Imaging 30: 1323–1341. 22770690 10.1016/j.mri.2012.05.001 PMC3466397

[bib33] Flint J. Eskin E., 2012 Genome-wide association studies in mice. Nat. Rev. Genet. 13: 807–817. 23044826 10.1038/nrg3335 PMC3625632

[bib34] Franchini P. Fruciano C. Spreitzer M. L. Jones J. C. Elmer K. R., 2013 Genomic architecture of ecologically divergent body shape in a pair of sympatric crater lake cichlid fishes. Mol. Ecol. 23: 1828–1845. 24237636 10.1111/mec.12590

[bib35] Galesloot T. E. van Steen K. Kiemeney L. A. L. M. Janss L. L. Vermeulen S. H., 2014 A Comparison of multivariate genome-wide association methods. PLoS One 9: e95923–e95928. 24763738 10.1371/journal.pone.0095923 PMC3999149

[bib36] Gao H. Zhang T. Wu Y. Wu Y. Jiang L., 2014 Multiple-trait genome-wide association study based on principal component analysis for residual covariance matrix. Heredity 113: 526–532. 24984606 10.1038/hdy.2014.57 PMC4274615

[bib37] Gnirke A. Melnikov A. Maguire J. Rogov P. LeProust E. M., 2009 Solution hybrid selection with ultra-long oligonucleotides for massively parallel targeted sequencing. Nat. Biotechnol. 27: 182–189. 19182786 10.1038/nbt.1523 PMC2663421

[bib38] Goodall C., 1991 Procrustes methods in the statistical analysis of shape. J. R. Stat. Soc. Series B Stat. Methodol. 53: 285–339.

[bib39] Gray M. M. Parmenter M. D. Hogan C. A. Ford I. Cuthbert R. J., 2015 Genetics of rapid and extreme size evolution in island mice. Genetics 201: 213–228. 26199233 10.1534/genetics.115.177790 PMC4566264

[bib40] Gunz P. Mitteroecker P., 2013 Semilandmarks: a method for quantifying curves and surfaces. Hystrix 24: 103–109.

[bib41] Gunz P. Mitteroecker P. Bookstein F. L., 2005 Semilandmarks in three dimensions, pp. 73–98 in Modern Morphometrics in Physical Anthropology, edited by Slice D. E. Kluwer Academic/Plenum Publishers, New York.

[bib42] Guo J. Mei X. Tang K., 2013 Automatic landmark annotation and dense correspondence registration for 3D human facial images. BMC Bioinformatics 14: 232. 23870191 10.1186/1471-2105-14-232 PMC3724574

[bib43] Hallgrímsson B. Mio W. Marcucio R. S. Spritz R., 2014 Let’s face it–complex traits are just not that simple. PLoS Genet. 10: e1004724. 25375250 10.1371/journal.pgen.1004724 PMC4222688

[bib44] Healy M. J. R., 1969 Rao’s paradox concerning multivariate tests of significance. Biometrics 25: 411–413.

[bib45] Hodges E. Xuan Z. Balija V. Kramer M. Molla M. N., 2007 Genome-wide in situ exon capture for selective resequencing. Nat. Genet. 39: 1522–1527. 17982454 10.1038/ng.2007.42

[bib46] Houle D. Govindaraju D. R. Omholt S., 2010 Phenomics: the next challenge. Nat. Rev. Genet. 11: 855–866. 21085204 10.1038/nrg2897

[bib47] Huang X. Feng Q. Qian Q. Zhao Q. Wang L., 2009 High-throughput genotyping by whole-genome resequencing. Genome Res. 19: 1068–1076. 19420380 10.1101/gr.089516.108 PMC2694477

[bib48] Huber B. Whibley A. Poul Y. L. Navarro N. Martin A., 2015 Conservatism and novelty in the genetic architecture of adaptation in Heliconius butterflies. Heredity 114: 515–524. 25806542 10.1038/hdy.2015.22 PMC4815517

[bib49] Jones M. R. Good J. M., 2016 Targeted capture in evolutionary and ecological genomics. Mol. Ecol. 25: 185–202. 26137993 10.1111/mec.13304 PMC4823023

[bib50] Khila A. Abouheif E. Rowe L., 2009 Evolution of a novel appendage ground plan in water striders is driven by changes in the Hox gene Ultrabithorax. PLoS Genet. 5: e1000583–e1000589. 19649305 10.1371/journal.pgen.1000583 PMC2709915

[bib51] Klingenberg C. P., 2010 Evolution and development of shape: integrating quantitative approaches. Nat. Rev. Genet. 11: 623–635. 20697423 10.1038/nrg2829

[bib52] Klingenberg C. P. Gidaszewski N. A., 2010 Testing and quantifying phylogenetic signals and homoplasy in morphometric data. Syst. Biol. 59: 245–261. 20525633 10.1093/sysbio/syp106

[bib53] Klingenberg C. P. Leamy L. J., 2001 Quantitative genetics of geometric shape in the mouse mandible. Evolution 55: 2342–2352. 11794792 10.1111/j.0014-3820.2001.tb00747.x

[bib54] Klingenberg, C. P., and N. Navarro, 2012 Development of the mouse mandible: a model system for complex morphological structures, pp. 135–149 in *Evolution of the House Mouse*, edited by Macholán, M., S. J. E. Baird, and J. Pialek. Cambridge University Press, Cambridge.

[bib55] Klingenberg C. P. Leamy L. J. Routman E. J. Cheverud J. M., 2001 Genetic architecture of mandible shape in mice: effects of quantitative trait loci analyzed by geometric morphometrics. Genetics 157: 785–802. 11156997 10.1093/genetics/157.2.785 PMC1461535

[bib56] Klingenberg C. P. Leamy L. J. Cheverud J. M., 2004 Integration and modularity of quantitative trait locus effects on geometric shape in the mouse mandible. Genetics 166: 1909–1921. 15126408 10.1534/genetics.166.4.1909 PMC1470826

[bib57] Krzanowski W. J., 1979 Between-Groups Comparison of Principal Components. J. Am. Stat. Assoc. 74: 703–707.

[bib58] Kwak I.-Y. Moore C. R. Spalding E. P. Broman K. W., 2014 A simple regression-based method to map quantitative trait loci underlying function-valued phenotypes. Genetics 197: 1409–1416. 24931408 10.1534/genetics.114.166306 PMC4125409

[bib59] Langlade N. B. Feng X. Dransfield T. Copsey L. Hanna A. I., 2005 Evolution through genetically controlled allometry space. Proc. Natl. Acad. Sci. USA 102: 10221–10226. 16009935 10.1073/pnas.0504210102 PMC1177394

[bib60] Leamy L. J. Klingenberg C. P. Sherratt E. Wolf J. B. Cheverud J. M., 2008 A search for quantitative trait loci exhibiting imprinting effects on mouse mandible size and shape. Heredity 101: 518–526. 18685568 10.1038/hdy.2008.79

[bib61] Ledur M. C. Navarro N. Pérez-Enciso M., 2009 Large-scale SNP genotyping in crosses between outbred lines: how useful is it? Heredity 105: 173–182. 19844266 10.1038/hdy.2009.149

[bib62] Linnen C. R. Poh Y.-P. Peterson B. K. Barrett R. D. H. Larson J. G., 2013 Adaptive evolution of multiple traits through multiple mutations at a single gene. Science 339: 1312–1316. 23493712 10.1126/science.1233213 PMC3836219

[bib63] Liu J. Gao W. Huang S. Nowinski W. L., 2008 A Model-Based, Semi-Global Segmentation Approach for Automatic 3-D Point Landmark Localization in Neuroimages. IEEE Trans. Med. Imaging 27: 1034–1044. 18672421 10.1109/TMI.2008.915684

[bib64] Liu J. Shikano T. Leinonen T. Cano J. M. Li M.-H., 2014 Identification of major and minor QTL for ecologically important morphological traits in three-spined sticklebacks (*Gasterosteus aculeatus*). G3 (Bethesda) 4: 595–604. 24531726 10.1534/g3.114.010389 PMC4059232

[bib65] Maga A. M. Navarro N. Cunningham M. L. Cox T. C., 2015 Quantitative trait loci affecting the 3D skull shape and size in mouse and prioritization of candidate genes in-silico. Front. Physiol. 6: 1–13. 25859222 10.3389/fphys.2015.00092 PMC4374467

[bib66] Manichaikul A. Dupuis J. Sen S. Broman K. W., 2006 Poor performance of bootstrap confidence intervals for the location of a quantitative trait locus. Genetics 174: 481–489. 16783000 10.1534/genetics.106.061549 PMC1569776

[bib67] Manichaikul A. Moon J. Y. Sen S. Yandell B. S. Broman K. W., 2009 A model selection approach for the identification of quantitative trait loci in experimental crosses, allowing epistasis. Genetics 181: 1077–1086. 19104078 10.1534/genetics.108.094565 PMC2651044

[bib68] McCane B., 2013 Shape variation in outline shapes. Syst. Biol. 62: 134–146. 22993142 10.1093/sysbio/sys080

[bib69] Mezey J. G. Houle D. Nuzhdin S. V., 2005 Naturally segregating quantitative trait loci affecting wing shape of Drosophila melanogaster. Genetics 169: 2101–2113. 15520257 10.1534/genetics.104.036988 PMC1449619

[bib70] Miller M. R. Dunham J. P. Amores A. Cresko W. A. Johnson E. A., 2007 Rapid and cost-effective polymorphism identification and genotyping using restriction site associated DNA (RAD) markers. Genome Res. 17: 240–248. 17189378 10.1101/gr.5681207 PMC1781356

[bib71] Monteiro L. R., 1999 Multivariate regression models and geometric morphometrics: the search for causal factors in the analysis of shape. Syst. Biol. 48: 192–199. 12078640 10.1080/106351599260526

[bib72] Mott R. Flint J., 2013 Dissecting quantitative traits in mice. Annu. Rev. Genomics Hum. Genet. 14: 421–439. 23834320 10.1146/annurev-genom-091212-153419

[bib73] Muñoz-Muñoz F. Perpiñan D., 2010 Measurement error in morphometric studies: comparison between manual and computerized methods. Ann. Zool. Fenn. 47: 46–56.

[bib74] Navarro, N., 2015 shapeQTL: shape QTL mapping experiment with R. R package version 0.2. https://github.com/nnavarro/shapeQTL. Accessed: January 28, 2016.

[bib75] Navarro, N., and C. P. Klingenberg, 2007 Mapping multiple QTLs of geometric shape of the mouse mandible, pp. 125–128 in *Systems Biology Statistical Bioinformatics*, edited by Barber, S., P. D. Baxter, and K. V. Mardia, Leeds University Press, Leeds.

[bib76] Olson C. L., 1974 Comparative Robustness of Six Tests in Multivariate Analysis of Variance. J. Am. Stat. Assoc. 69: 894–908.

[bib77] Olson C. L., 1976 On choosing a test statistic in multivariate analysis of variance. Psychol. Bull. 83: 579–586.

[bib78] Olson M., 2007 Enrichment of super-sized resequencing targets from the human genome. Nat. Methods 4: 891–892. 17971778 10.1038/nmeth1107-891

[bib79] Pallares L. F. Harr B. Turner L. M. Tautz D., 2014 Use of a natural hybrid zone for genomewide association mapping of craniofacial traits in the house mouse. Mol. Ecol. 23: 5756–5770. 25319559 10.1111/mec.12968

[bib80] Pallares L. F. Carbonetto P. Gopalakrishnan S. Parker C. C. Ackert-Bicknell C. L., 2015 Mapping of craniofacial traits in outbred mice identifies major developmental genes involved in shape determination. PLoS Genet. 11: e1005607. 26523602 10.1371/journal.pgen.1005607 PMC4629907

[bib81] Perakis, P., G. Passalis, T. Theoharis, and I. A. Kakadiaris, 2010 3D facial landmark detection & face registration, Tech. Rep., University of Athens, Greece.

[bib82] Perakis P. Theoharis T. Kakadiaris I. A., 2014 Feature fusion for facial landmark detection. Pattern Recognit. 47: 2783–2793.

[bib83] Percival C. J. Liberton D. K. Pardo-Manuel de Villena F. Spritz R. Marcucio R., 2016 Genetics of murine craniofacial morphology: diallel analysis of the eight founders of the Collaborative Cross. J. Anat. 228: 96–112. 26426826 10.1111/joa.12382 PMC4694168

[bib84] Peterson B. K. Weber J. N. Kay E. H. Fisher H. S. Hoekstra H. E., 2012 Double digest RADseq: an inexpensive method for de novo SNP discovery and genotyping in model and non-model species. PLoS One 7: e37135. 22675423 10.1371/journal.pone.0037135 PMC3365034

[bib85] Prud’homme B. Minervino C. Hocine M. Cande J. D. Aouane A., 2012 Body plan innovation in treehoppers through the evolution of an extra wing-like appendage. Nature 473: 83–86. 21544145 10.1038/nature09977

[bib86] R Core Team, 2015 R: A Language and Environment for Statistical Computing. R Foundation for Statistical Computing, Vienna, Austria. URL http://www.R-project.org/.

[bib87] Rolfe S. M. Shapiro L. G. Cox T. C. Maga A. M. Cox L. L., 2011 A landmark-free framework for the detection and description of shape differences in embryos. Conf. Proc. IEEE Eng. Med. Biol. Soc. 2011: 5153–5156. 22255499 10.1109/IEMBS.2011.6091276 PMC3261520

[bib88] Schlager, S., 2015a Morpho: Calculations and visualisations related to Geometric Morphometrics. R-package version 2. 3.0. Available at: https://github.com/zarquon42b/Morpho. Accessed: September 10, 2015

[bib89] Schlager S., 2015 b Rvcg: Manipulations of Triangular Meshes Based on the ’VCGLIB’ API. Available at: https://github.com/zarquon42b/Rvcg. Accessed: September 10, 2015

[bib90] Sen S. Churchill G. A., 2001 A statistical framework for quantitative trait mapping. Genetics 159: 371–387. 11560912 10.1093/genetics/159.1.371 PMC1461799

[bib91] Shifman S. Bell J. T. Copley R. R. Taylor M. S. Williams R. W., 2006 A high-resolution single nucleotide polymorphism genetic map of the mouse genome. PLoS Biol. 4: e395. 17105354 10.1371/journal.pbio.0040395 PMC1635748

[bib92] Stephens M., 2013 A unified framework for association analysis with multiple related phenotypes. PLoS One 8: e65245. 23861737 10.1371/journal.pone.0065245 PMC3702528

[bib93] Suto J.-I., 2009 Identification of multiple quantitative trait loci affecting the size and shape of the mandible in mice. Mamm. Genome 20: 1–13. 19067046 10.1007/s00335-008-9154-5

[bib94] Swiderski D. L. Zelditch M. L., 2013 The complex ontogenetic trajectory of mandibular shape in a laboratory mouse. J. Anat. 223: 568–580. 24111948 10.1111/joa.12118 PMC3842199

[bib95] Tabachnick B. G. Fidell L. S., 2013 Using Multivariate Statistics, Ed. 6th Pearson Education, Inc, Boston.

[bib96] Teplitsky C. Robinson M. R. Merilä J., 2014 Evolutionary potential and constraints in wild populations, pp. 190–208 in Quantitative Genetics in the Wild, edited by Charmantier A. Garant D. Kruuk L. E. B. Oxford University Press, Oxford.

[bib97] Valdar W. Solberg L. C. Gauguier D. Burnett S. Klenerman P., 2006 Genome-wide genetic association of complex traits in heterogeneous stock mice. Nat. Genet. 38: 879–887. 16832355 10.1038/ng1840

[bib98] Workman M. S. Leamy L. J. Routman E. J. Cheverud J. M., 2002 Analysis of quantitative trait locus effects on the size and shape of mandibular molars in mice. Genetics 160: 1573–1586. 11973311 10.1093/genetics/160.4.1573 PMC1462040

[bib99] Yalcin B. Nicod J. Bhomra A. Davidson S. Cleak J., 2010 Commercially available outbred mice for genome-wide association studies. PLoS Genet. 6: e1001085. 20838427 10.1371/journal.pgen.1001085 PMC2932682

[bib100] Yezerinac S. M. Lougheed S. C. Handford P., 1992 Measurement error and morphometric studies: statistical power and observer experience. Syst. Biol. 41: 471–482.

[bib101] Young R. Maga A. M., 2015 Performance of single and multi-atlas based automated landmarking methods compared to expert annotations in volumetric microCT datasets of mouse mandibles. Front. Zool. 12: 33. 26628903 10.1186/s12983-015-0127-8 PMC4666065

[bib102] Zeng Z. B. Liu J. Stam L. F. Kao C. H. Mercer J. M., 2000 Genetic architecture of a morphological shape difference between two Drosophila species. Genetics 154: 299–310. 10628989 10.1093/genetics/154.1.299 PMC1460924

