# Supplementary material for: Does 3D Phenotyping Yield Substantial Insights in the Genetics of the Mouse Mandible Shape?
Source: G3 (Bethesda). 2016 Feb 23;6(5):1153–63. doi: 10.1534/g3.115.024372 (PMC4856069; doi:10.1534/g3.115.024372)
Supplement: Supporting Information [file supp_6_5_1153__index.html]

Does 3D Phenotyping Yield Substantial Insights in the Genetics of the Mouse Mandible Shape? — Supporting Information 

# Does 3D Phenotyping Yield Substantial Insights in the Genetics of the Mouse Mandible Shape?

## Supporting Materials for Navarro and Maga, 2016

**Files in this Data Supplement:**

- Figure S1 - Visualization of shape changes associated with QTLs from 3D manual landmarks. (.pdf, 18017 KB)
- Figure S2 - Visualization of shape changes associated with QTLs from semilandmarks. (.pdf, 24139 KB)
- Table S1 - Effect sizes of covariates. (.pdf, 61 KB)
- Table S2 - QTL positions, replications, effect sizes and proportion along *z*. (.pdf, 101 KB)
- File S1 - Genotypes and phenotypes are available as a cross object readable by R/qtl or R/shapeQTL. (.zip, 3324 KB)
