# Supplementary material for: Does 3D Phenotyping Yield Substantial Insights in the Genetics of the Mouse Mandible Shape?
Source: G3 (Bethesda). 2016 Feb 23;6(5):1153–63. doi: 10.1534/g3.115.024372 (PMC4856069; doi:10.1534/g3.115.024372)
Supplement: Supporting Information [file supp_g3.115.024372_FigureS2.pdf]

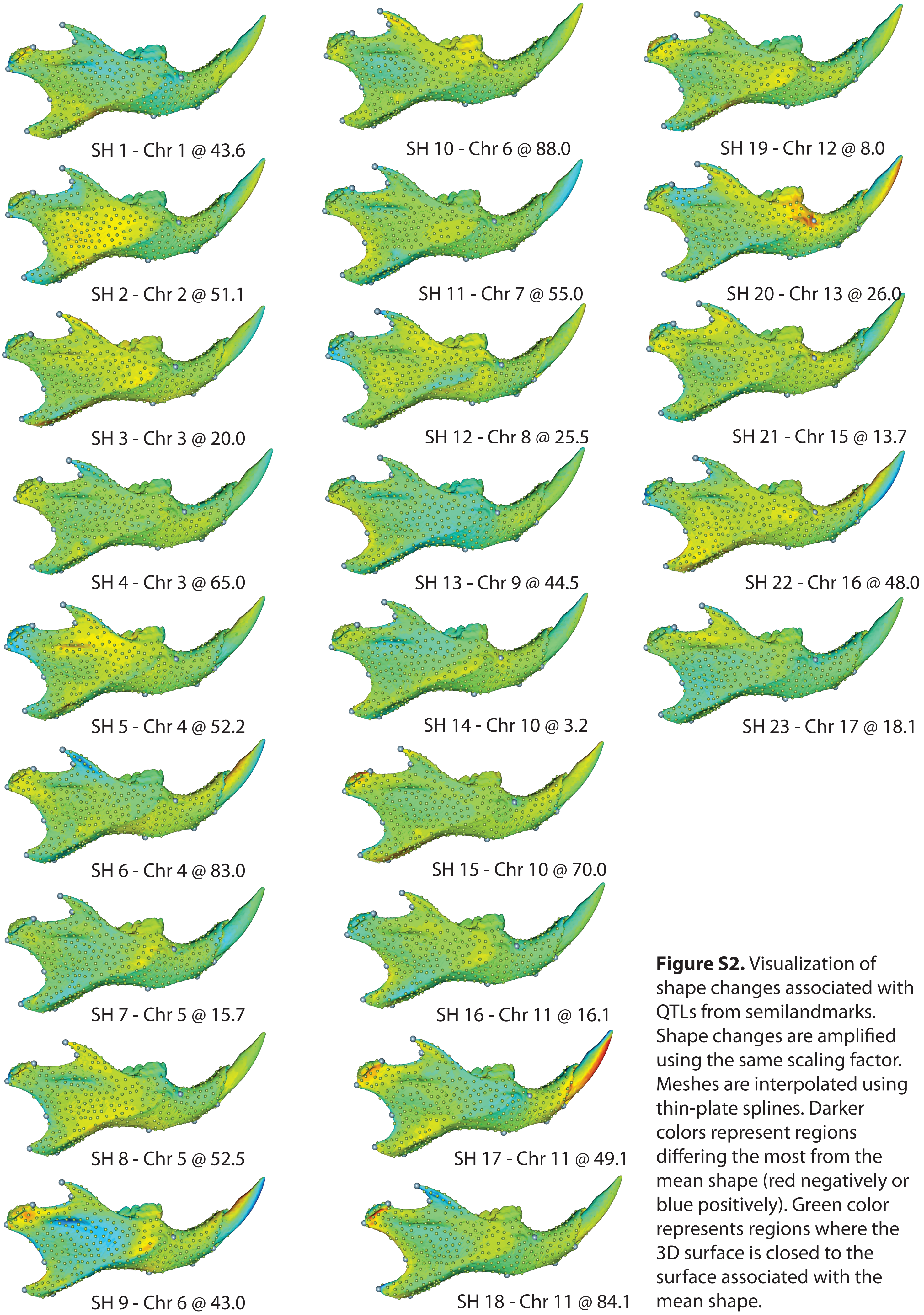

Figure S2. Visualization of shape changes associated with QTLs from semilandmarks. Shape changes are amplified using the same scaling factor. Meshes are interpolated using thin-plate splines. Darker colors represent regions differing the most from the mean shape (red negatively or blue positively). Green color represents regions where the 3D surface is closed to the surface associated with the mean shape.
