# Supplementary material for: Does 3D Phenotyping Yield Substantial Insights in the Genetics of the Mouse Mandible Shape?
Source: G3 (Bethesda). 2016 Feb 23;6(5):1153–63. doi: 10.1534/g3.115.024372 (PMC4856069; doi:10.1534/g3.115.024372)
Supplement: Supporting Information [file supp_g3.115.024372_TableS1.pdf]

Table S1. Effect sizes of covariates

Dataset	Effect	$\|\beta\|$ [1]	% SST[2]	% SS proj. Scores[3]
**2D**	logCS	0.134	1.48	18.66 (7.9)
	dir.Cross	0.009	2.49	25.39 (9.8)
	gender	0.005	0.85	13.81 (6.2)
**3D**	logCS	0.132	1.31	24.09 (5.4)
	dir.Cross	0.009	2.46	26.76 (9.2)
	gender	0.005	0.85	14.04 (6.0)
**Semiland**	logCS	0.142	1.85	31.04 (6.0)
	dir.Cross	0.008	2.79	36.17 (7.7)
	gender	0.005	0.94	18.81 (5.0)

[1] the magnitude of the effect in unit of Procrustes distances. It correspond to the sum of squared elements

[2] the percentage of SST is the percentage of total Procrustes variance explained by the covariate effect

[3] the percentage of variance accounted for in the specific direction defined by the $\boldsymbol{\beta}$ vector. It corresponds to the projection of the dispersion matrix of the fitted values on the projection of the total dispersion. In parenthesis, the percentage of total Procrustes variance in that direction (i.e, the effect plus the error) is reported
