# Supplementary material for: Does 3D Phenotyping Yield Substantial Insights in the Genetics of the Mouse Mandible Shape?
Source: G3 (Bethesda). 2016 Feb 23;6(5):1153–63. doi: 10.1534/g3.115.024372 (PMC4856069; doi:10.1534/g3.115.024372)
Supplement: Supporting Information [file supp_g3.115.024372_TableS2.pdf]

Table S2. QTL positions, replications, effect sizes and proportion along *z*

QTL	mrk	Chr	Left	Pos	Right	2D	3D	Semi	% SST[1]	% SS proj Scores[1]	% z[1]
**SH1_2D**	gnf01.075.385	1	41.33	43.62	45.33		SH1_3D	SH1_Semi	0.83	6.53	
**SH2_2D**	rs3667007	2	49.54	51.54	60.54		SH3_3D	SH2_Semi	1.14	11.59	
**SH3_2D**	rs13477217	3	12.01	35.86	60.89		SH4_3D		0.88	9.16	
**SH4_2D**	rs6226080	4	56.01	66.01	76.01		SH5_3D		1.09	7.56	
**SH5_2D**	rs3680434	5	10.5	16.5	19.5		SH6_3D	SH7_Semi	0.54	5.72	
**SH6_2D**	rs13478337	5	47.5	51.5	80.5		SH7_3D	SH8_Semi	0.70	6.74	
**SH7_2D**	CEL-6_86289708	6	30.00	42.00	44.00		SH8_3D	SH9_Semi	0.88	8.42	
**SH8_2D**	rs6386110	8	17.21	23.52	25.52		SH9_3D	SH12_Semi	1.45	9.5	
**SH9_2D**	rs3655717	9	17.1	39.1	72.1		SH10_3D	SH13_Semi	0.61	7.21	
**SH10_2D**	rs13480773	10	59.03	66.03	77.03		SH11_3D	SH15_Semi	0.59	7.87	
**SH11_2D**	rs13481109	11	45.08	47.08	49.08		SH12_3D	SH17_Semi	0.94	5.81	
**SH12_2D**	rs3672597	11	81.08	83.13	86.08		SH13_3D	SH18_Semi	0.69	8.17	
**SH13_2D**	rs6311081	12	11.9	16.9	19.98		SH14_3D		0.77	5.18	
**SH14_2D**	rs3720782	13	19.00	26.52	26.52		SH15_3D	SH20_Semi	0.78	7.5	
**SH15_2D**	CEL-15_36490596	15	12.99	13.68	17.99		SH17_3D	SH21_Semi	0.72	8.19	
**SH16_2D**	rs4204106	16	44.01	48.03	50.03		SH18_3D	SH22_Semi	0.79	6.96	
**SH17_2D**	rs6229946	17	37.14	47.14	57.14		SH19_3D		0.84	7.33	
**SH1_3D**	rs13475866	1	25.06	27.33	44.33	SH1_2D		SH1_Semi	0.56	4.17	0.11
**SH2_3D**	rs6157620	1	84.33	101.84	105.33				0.62	5.85	0.32
**SH3_3D**	rs6316774	2	47.54	57.08	62.85	SH2_2D		SH2_Semi	0.93	8.76	0.13
**SH4_3D**	rs13477217	3	31.01	35.86	40.01	SH3_2D			0.85	6.47	0.07
**SH5_3D**	rs13477838	4	52.01	54.01	64.01	SH4_2D		SH5_Semi	1.05	6.67	0.15
**SH6_3D**	rs3680434	5	10.5	16.5	21.5	SH5_2D		SH7_Semi	0.58	5.00	0.08
**SH7_3D**	rs13478337	5	47.5	51.5	55.5	SH6_2D		SH8_Semi	0.71	5.88	0.09
**SH8_3D**	CEL-6_83434907	6	34.00	40.4	43.00	SH7_2D		SH9_Semi	0.91	7.46	0.18
**SH9_3D**	rs6386110	8	17.21	24.52	46.52	SH8_2D		SH12_Semi	1.25	7.15	0.04
**SH10_3D**	rs3655717	9	36.1	39.1	82.1	SH9_2D		SH13_Semi	0.59	6.33	0.11
**SH11_3D**	rs13480740	10	59.03	61.03	67.03	SH10_2D		SH15_Semi	0.63	5.16	0.27
**SH12_3D**	rs13481109	11	45.08	47.08	49.08	SH11_2D		SH17_Semi	0.92	5.52	0.07

**SH13_3D**	rs3672597	11	81.08	83.13	86.08	SH12_2D		SH18_Semi	0.63	6.38	0.08
**SH14_3D**	UT_12_24.561109	12	8.9	12.9	19.98	SH13_2D		SH19_Semi	0.69	4.44	0.10
**SH15_3D**	rs3720782	13	23.00	26.52	26.52	SH14_2D		SH20_Semi	0.98	7.28	0.22
**SH16_3D**	rs6396829	14	8.99	11.99	14.99				0.51	5.52	0.26
**SH17_3D**	CEL-15_43206205	15	12.99	15.99	17.4	SH15_2D		SH21_Semi	0.8	6.82	0.11
**SH18_3D**	rs4204106	16	43.03	48.03	51.03	SH16_2D		SH22_Semi	0.78	6.35	0.10
**SH19_3D**	rs13483135	17	17.14	56.14	58.49	SH17_2D			0.85	7.14	0.06
**SH1_Semi**	gnf01.075.385	1	42.33	43.62	44.33	SH1_2D	SH1_3D		0.91	5.6	0.11
**SH2_Semi**	rs3722345	2	51.09	51.09	60.54	SH2_2D	SH3_3D		0.85	8.62	0.17
**SH3_Semi**	rs6274061	3	12.01	20.01	21.01				0.67	5.82	0.13
**SH4_Semi**	rs3676039	3	59.01	65.01	77.01				0.31	5.11	0.19
**SH5_Semi**	rs3711477	4	52.01	52.2	53.01		SH5_3D		0.63	5.10	0.20
**SH6_Semi**	UT_4_132.137715	4	81.01	83.01	84.01				0.70	6.30	0.13
**SH7_Semi**	rs13478154	5	13.5	15.71	17.5	SH5_2D	SH6_3D		0.70	7.16	0.16
**SH8_Semi**	rs13478388	5	43.5	52.5	56.5	SH6_2D	SH7_3D		0.60	5.31	0.13
**SH9_Semi**	CEL-6_86289708	6	41.55	43.00	43.00	SH7_2D	SH8_3D		1.09	4.92	0.20
**SH10_Semi**	rs3658783	6	84.00	88.00	89.28				0.40	5.09	0.21
**SH11_Semi**	rs13479427	7	43.05	55.02	57.2				0.54	5.96	0.08
**SH12_Semi**	rs6386110	8	22.38	25.52	27.52	SH8_2D	SH9_3D		1.01	6.76	0.11
**SH13_Semi**	rs3721056	9	43.1	44.47	71.1	SH9_2D	SH10_3D		0.61	4.48	0.20
**SH14_Semi**	rs3686911	10	3.03	3.18	9.03				0.53	6.42	0.13
**SH15_Semi**	mCV24217147	10	67.03	70.03	71.12	SH10_2D	SH11_3D		0.58	4.84	0.17
**SH16_Semi**	rs3700830	11	12.08	16.08	17.08				0.35	4.38	0.29
**SH17_Semi**	rs13481127	11	48.08	49.08	54.08	SH11_2D	SH12_3D		0.6	4.81	0.21
**SH18_Semi**	rs3672597	11	82.08	84.08	86.08	SH12_2D	SH13_3D		0.53	4.33	0.13
**SH19_Semi**	rs13481321	12	6.9	7.99	8.95		SH14_3D		0.84	6.21	0.13
**SH20_Semi**	rs3693942	13	25.00	26.00	26.52	SH14_2D	SH15_3D		1.01	5.94	0.11
**SH21_Semi**	CEL-15_36490596	15	13.68	13.68	14.99	SH15_2D	SH17_3D		0.67	6.24	0.10
**SH22_Semi**	rs4204106	16	33.03	48.03	53.31	SH16_2D	SH18_3D		1.10	8.97	0.07
**SH23_Semi**	rs6298471	17	16.03	18.14	21.14				0.59	6.64	0.12

[1] See Table S1 and the main text for an explanation of these percentage of variance explained.
